# Narcissism and Affective Polarization

**DOI:** 10.1007/s11109-024-09963-5

**Published:** 2024-08-02

**Authors:** James Tilley, Sara Hobolt

**Affiliations:** 1 University of Oxford, Oxford, UK; 2 London School of Economics and Political Science, London, UK

**Keywords:** Affective polarization, Partisanship, Political identity, Personality, Narcissism

## Abstract

There are increasing concerns about affective polarization between political groups in the US and elsewhere. While most work explaining affective polarization focuses on a combination of social and ideological sorting, we ask whether people’s personalities are associated with friendliness to their political in-group and hostility to their political out-group. We argue that the personality trait of narcissism (entitled self-importance) is an important correlate of affective polarization. We test this claim in Britain using nationally representative survey data, examining both long-standing party identities and new Brexit identities. Our findings reveal that narcissism, and particularly the ‘rivalry’ aspect of narcissism, is associated with both positive and negative partisanship. This potentially not only explains why some people are more susceptible to affective polarization, but also has implications for elite polarization given that narcissism is an important predictor of elite entry.

**Supplementary Information:**

The online version contains supplementary material available at 10.1007/s11109-024-09963-5.

A well-functioning democracy requires that citizens, and politicians, are willing to engage respectfully with each other and ultimately to compromise (Lipset, 1959; McCoy et al., 2018; Strickler, 2018). Affective polarization, entrenched political in-group identities accompanied by hostility towards the out-group, instead brings intolerance, political cynicism and dissension (Hetherington & Rudolph, 2015; Layman et al., 2006). The increased affective polarization of politics in the US over the last few decades (Iyengar & Westwood, 2015; Iyengar et al., 2012, 2019; Mason, 2018) has therefore caused much disquiet. Yet, recent comparative research has shown that affective polarization is equally pronounced in many other countries (Gidron et al., 2020, 2023; Harteveld, 2021a; Huddy et al., 2018; Reiljan, 2020; Wagner, 2021) and that it is not even confined to party identities. In Britain, for example, the heated Brexit referendum and aftermath saw the emergence of two new political tribes of Remainers and Leavers with the same in-group and out-group tensions that we associate with partisanship (Hobolt et al., 2021; Tilley & Hobolt, 2023).

The causes of affective polarization are normally related to either ideological or social sorting or both (Levendusky, 2009; Harteveld, 2021b; Mason, 2015, 2018). These social group and ideological factors are clearly important drivers of affective polarization, but there is less research on the psychological characteristics of those who are affectively polarized and thus the traits which people on both sides of the ideological divide may have in common. In this article, we suggest that the specific personality trait of narcissism could be an explanation for why some people become affectively polarized.

We build on recent work which examines the role of narcissism in political attitudes and vote choice (Hart & Stekler, 2022; Hatemi & Fazekas, 2018; Mayer et al., 2020). But rather than examining the *differences* between groups (Republicans and Democrats, or liberals and conservatives), we argue that there are clear *similarities* in the personalities of people who are affectively polarized. Specifically, people who are higher in the personality trait of narcissism are more likely to be affectively polarized. Narcissism has at its heart an emphasis on ‘entitled self-importance’ (Krizan & Herlache, 2018, p. 6) and thus affects how people see both in-groups and out-groups. Since ‘narcissism serves as an important component of identity regulation that results in positive feelings about the self and groups they belong to’ (Hatemi & Fazekas, 2018, p. 874), we expect that people higher in narcissism will have stronger in-group political identities. Equally, that sense of entitlement and superiority should also lead people high in narcissism to be more hostile towards the political out-group given the out-group’s perceived lack of deservingness.

We test our expectations using original, nationally representative survey data from Britain. This allows us to examine both long-standing party identities and newer Brexit identities, both of which were salient and strongly held political identities at the time of our study (Hobolt & Tilley, 2021; Hobolt et al., 2021). Importantly, we disaggregate affective polarization into two elements: an emotionally resonant in-group identity and a hostility towards those with an out-group identity. By separately looking at in-group affinity and out-group animosity, we are able to show the different potential effects of narcissism on the twin underlying processes that generate affective polarization. We also disaggregate narcissism itself into two lower-level personality aspects: admiration (superiority and self-importance) and rivalry (antagonistic entitlement).

Overall, our findings show that people high in narcissism, and particularly rivalry, are more likely to be affectively polarized regardless of their identity type and their specific in-group. Thus, although the affectively polarized may regard the ‘other side’ as the enemy, they, in fact, do have something in common. This matters, not only because it highlights some of the similarities of affectively polarized people on both sides of a political divide, but also because personality traits, such as narcissism, have been shown to be important predictors of political behavior. In particular, since people entering politics tend to be high in narcissism (Blais & Pruysers, 2017; Peterson & Palmer, 2022; Post, 2015), our findings may help explain why political elites often appear more affectively polarized than the average voter.

## Affective Polarization

While political conflict is central to democratic societies, it is worrisome when such conflict solidifies and political identities crystallize into polarized groups. In recent decades, there has been increasing partisan polarization in American politics (Layman et al., 2006; Mason, 2018). This means not just strong in-group attachments to parties, but also interpersonal animosity across party lines, with Democrats and Republicans increasingly expressing dislike for one another (Iyengar & Westwood, 2015; Iyengar et al., 2012; Mason, 2015, 2018). This phenomenon has been described as *affective polarization*: an emotional attachment to in-group partisans and hostility towards out-group partisans (Iyengar & Westwood, 2015; Iyengar et al., 2012, 2019; Kingzette et al., 2021).

What explains affective polarization? On the one hand, some have argued that as partisan identities in the US have become increasingly aligned with other group identities, such as race and religion, levels of in-party affinity and out-party hostility have grown (Harteveld, 2021b; Iyengar et al., 2012; Mason, 2015, 2018). A lack of cross-cutting group identities intensifies both the emotional attachment to the in-group and the hostility to the out-group, as it is easier for partisans to make generalized inferences about the ‘other side’ (Mason, 2018). On the other hand, there is also an obvious role for ideological polarization as a cause of affective polarization (Dias & Lelkes, 2022). As Webster and Abramowitz (2017, p. 643) show, there is ‘clear evidence that there is a causal relationship between ideological distance and affect’ which implies that change may also be due to mass ideological polarization. Both of these processes, in different ways, have then been aided by polarization among US elites and partisans responding to elite cues (Banda & Cluverius, 2018; Lelkes, 2021; Rogowski & Sutherland, 2016), and a polarized US media environment that activates partisan identities (Lelkes et al., 2017; Levendusky, 2013; Suhay et al., 2018).

Yet, Americans are far from unique when it comes to strong partisan in-group attachments and out-group partisan hostility and the processes which generate them (Gidron et al., 2023; Harteveld, 2021a, 2021b; Reiljan, 2020; Wagner, 2021). In their comprehensive comparative study, Gidron et al. (2020) find high levels of affective polarization outside the US and there is mounting evidence for affective polarization among other non-partisan political groups. For example, Hobolt et al. (2021) show that affective polarization emerged along the Brexit fault line in the context of the UK’s highly divisive referendum on EU membership in 2016. This was very similar in type to partisan polarization (strong in-group affinity and out-group hostility), but based on the referendum rather than driven by political parties.

This research has clearly advanced our understanding of political identity groups and affective polarization in the US and around the world. Yet we know much less about the individual psychological traits that make some people, regardless of their social group and ideological position, more likely to engage in affective polarization. This question is especially important given the well-established relevance of personality traits in explaining individual-level differences in political behavior. In this paper, we thus shift the focus to ask whether personality characteristics affect the degree to which individuals become affectively polarized.

## Personality and Affective Polarization

How might personality traits affect political in-group identity attachment and political out-group animosity? There is no shortage of research about personality and politics. Most of this research concentrates on the Big Five personality traits developed by McCrae and Costa (2003; see McCrae, 2009 for an excellent summary). Associations between the Big Five personality traits and various political attitudes have been studied for several decades. The main stylized fact that emerges from this research is that people who score higher on openness to experience tend to have more left-wing views, whereas those who score higher on conscientiousness tend to hold more right-wing views (Gerber et al., 2010, 2011; Johnston et al., 2017; Mondak, 2010). A less examined question is how personality shapes attachment to political identities in general. Two important exceptions are Gerber et al. (2012) and Bakker et al. (2015) who show that extraversion correlates with measures of partisan strength. These associations are typically assumed to reflect a causal relationship between personality and politics given personality is seen in these accounts as largely fixed after childhood. This assumption has been criticized recently (Bakker et al., 2021; Boston et al., 2018), and we discuss this point further in the conclusion. For now, we assume a potential causal path from personality to politics.

This previous work focuses on the Big Five and political identities, but it does not capture one important personality trait: narcissism. The connection between narcissism and political behavior, above and beyond the Big Five, has only been recognized relatively recently. One of the so-called ‘Dark Triad’ (the other two being Machiavellianism and psychopathy), narcissism has been convincingly linked to ideology (Hatemi & Fazekas, 2018), support for particular parties (Mayer et al., 2020), support for democracy (Marchlewska et al., 2019), belief in conspiracy theories (Cichocka et al., 2016), political ambition (Blais & Pruysers, 2017; Hart et al., 2022; Peterson & Palmer, 2022), and political participation and interest (Chen et al., 2020; Fazekas & Hatemi, 2021). Of particular interest to the study of affective polarization is recent work in psychology that relates narcissism to prejudice towards out-groups. As people high in narcissism seek power, control and status, they tend to be more prejudiced towards low-status groups such as ethnic minorities and refugees (Jonason et al., 2020; Żemojtel-Piotrowska et al., 2020; although also see Anderson and Cheers (2018) who find that narcissism is unrelated to prejudice). One mechanism here is via the positive association between narcissism and social dominance orientation (Cichocka et al., 2017). Overall, this suggests that there may be a link between narcissism and views of political in- and out-groups.

Narcissism is a concept that even lower level aspects of the Big Five do not fully capture (Back et al., 2013; Leckelt et al., 2018).^1^ Although there remains some debate about what narcissism should encompass, and therefore how to measure it, we rely here on Krizan and Herlache’s (2018) overview of the narcissism literature and their advocacy of ‘entitled self-importance’ as the central idea. Importantly, this relates to the notion of ‘grandiose’ narcissism rather than ‘vulnerable’ narcissism (e.g., defensiveness, withdrawal and resentment).^2^ According to this conceptualization, narcissism is at its core about self-importance, the degree to which people think they are better than others, and entitlement, the degree to which people think they deserve more than others. Although there are multiple ways to operationalize the overall concept,^3^ we use the Narcissistic Admiration and Rivalry Questionnaire (NARQ) developed by Back et al. (2013). Of the measures which Krizan and Herlache (2018) review, this captures ‘entitled self-importance’ most thoroughly since the concept, and measure, have two underlying aspects of admiration and rivalry. The admiration aspect is most associated with self-importance and superiority and the rivalry aspect focuses on entitlement.

Clearly, the ideas of superiority and deservingness that underpin the trait of narcissism could be important when it comes to affective polarization. As Krizan and Herlache (2018, p. 19) state when discussing the Back et al. (2013) concept, the ‘narcissistic quest for self-enhancement (i.e. maintaining a grandiose self) takes two main forms: assertive self-enhancement and antagonistic self-protection’. An affinity for one’s political in-group is assertive self-enhancement and animosity towards one’s political out-group is antagonistic self-protection. Together they form affective polarization. Empirically, we know that narcissism predicts inter-group aggressiveness and out-group prejudice driven by perceived threats to the in-group (Golec de Zavala, 2011; Golec de Zavala et al., 2013) and a desire for social dominance (Cichocka et al., 2017; Jonason et al., 2020). Equally, political science research has shown that ‘perceptions of competition and threat’ are central to how we see inter-party relations (Satherley et al., 2020, p. 10).

Thus, there is good reason to think that narcissism and affective polarization may be related. It is worth noting that the existing research on personality and affective polarization also hints at this potential connection, given the focus on the Big Five trait of agreeableness^4^ and polarization. This is because agreeableness is negatively correlated with narcissism (Back et al., 2013; Leckelt et al., 2018).^5^ For example, Crawford and Brandt (2019) show that agreeableness affects prejudice across different, including political, out-groups and Webster (2018) finds that higher levels of agreeableness tend to reduce negative affect towards the out-group conditional on being a negative partisan. Hypothesis 1 is thus straightforward. We expect narcissism to positively correlate with affective polarization.

### H1

Higher levels of narcissism are associated with higher levels of affective polarization.

We also have expectations about which elements of affective polarization should be most associated with narcissism. In particular, we expect that there should be a stronger connection with out-group animosity, or negative partisanship, than with in-group affinity, or positive partisanship. While narcissism encompasses vanity and self-admiration, this admiration tends to focus on the self, not the group. The mechanism is thus indirect: contagion from self-regard to group-regard. By contrast, the other element of narcissism is entitlement which has, at its heart, a direct emphasis on others, and especially people in out-groups, being inferior and a potential threat.

### H2

Narcissism is more strongly associated with out-group animosity than in-group affinity.

As mentioned, we rely on a measure of narcissism (NARQ) which allows us to break down further the mechanisms that link affective polarization with narcissism. This is because there are two core aspects which underpin the concept of narcissism measured by the NARQ: admiration and rivalry. As Back et al. (2013) state, the former is focused on ‘grandiose self and charming self-assured behaviors’ (p. 1014) ‘to approach social admiration by means of self-promotion’ (p. 1015), but the latter is based on ‘devaluation of others and hostile aggressive behaviors’ (p. 1014) ‘to prevent social failure by means of self-defence’ (p. 1015). We thus hypothesize that the two aspects of narcissism, *rivalry* and *admiration*, will both be related to affective polarization since group competition will be more attractive and interesting to those who think themselves superior (high in admiration) and those who are more entitled and antagonistic (high in rivalry). Nonetheless, we also hypothesize that rivalry will be the dominant aspect. Rivalry should dominate, because it is this antagonistic entitlement focused on self-defense that should create direct hostility to the out-group but also mean that people cling more strongly to their in-group due to out-group threat.

### H3

Rivalry and admiration are both associated with affective polarization, but rivalry has a stronger association.

In this paper, we thus aim to assess fully how narcissism, and its twin components, relate to affective polarization. We examine the links to both in-group affinity and out-group animosity by carefully distinguishing between positive and negative attachments to political identities and positive and negative assessments of in- and out-groups. We also replicate all our work with not just party identity, but also Brexit identity: a political identity based on people’s 2016 EU referendum vote.

## Data and Measurement

### Data

Our data come from a nationally representative two-wave panel survey conducted by YouGov in Britain. Using the British case allows us to examine whether we find similar relationships between narcissism and affective polarization across the two salient political identities at the time: long-standing party identities and more recent Brexit identities. The first wave of the survey in March 2021 sampled 3,552 respondents and included a battery of personality questions. We then re-interviewed respondents in July 2021 and asked the questions that form our dependent variables. 2,017 respondents completed the second wave of the survey giving a 77% retention rate from wave 1 to wave 2. Our design means that we separate the measures of personality and politics, which helps to ameliorate the immediate cueing of political opinions after completing the long personality trait batteries of items.^6^ Nonetheless, as our data is still fundamentally cross-sectional, we discuss our results in terms of association.

### Measuring Identities

We first divide our sample into identity groups. 78% of people held a party identity: 35% Conservative, 24% Labour, 9% Liberal Democrats and 10% another party. Slightly fewer (75%) held a Brexit identity and this is fairly evenly split between Leavers (35%) and Remainers (40%). Online Appendix 1 shows the full descriptive statistics. It is worth noting that the two identities overlap to a certain extent. Nonetheless, 37% of Leavers are not Conservatives, 60% of Remainers are not Labour partisans and 57% of people who do not have a Brexit identity do have a partisan identity. These are related identities, but far from identical.

### Measuring Affective Polarization

As discussed, we break down affective polarization into two core elements: one focuses on in-group affinity and one on out-group animosity. While the literature often combines measures of in-group affinity with out-group animosity we measure these features separately using in-group and out-group question batteries. These allow us to capture affect towards both parties and partisans (Druckman & Levendusky, 2019; Kingzette, 2021).

For in-group affinity, we measure people’s emotional attachment to their political in-group identity (or positive partisanship) using a battery of five questions (Greene, 2000; Huddy et al., 2015). We ask people, with regard to their in-group, whether they agree or disagree with the following statements:

When I speak about the [in-group], I usually say ‘we’ instead of ‘they’

When people criticize the [in-group], it feels like a personal insult

I have a lot in common with other supporters of the [in-group]

When I meet someone who supports the [in-group], I feel connected with this person

When people praise the [in-group], it makes me feel good

The response options were ‘strongly disagree’ to ‘strongly agree’ which are scored 1–5 and then averaged. Alpha scores are above 0.8 and factor analyses show one factor solutions for all identity types. Full descriptive statistics are in Online Appendix 2.

We also replicate measures of stereotypes used by Iyengar et al. (2012). Specifically, we ask people how well they thought two positive characteristics (honesty and intelligence) and two negative characteristics (selfishness and hypocrisy) describe Leavers, Remainers, Conservative supporters and Labour supporters.^7^ Agreement is on a 1–5 scale from 1 (not at all well) to 5 (very well). We combine the four items, reversing the negative characteristic scores, to make an additive scale, running from 1–5, that measures positive perceptions of the respondents’ in-group. Alpha scores vary from 0.64 (Conservative partisans’ views of fellow Conservatives) to 0.79 (Remainers’ views of fellow Remainers).

The second element of polarization is out-group hostility. We again measure this in two ways. Our first measure was only asked of partisans and uses a battery of items designed to measure negative partisanship based on Bankert (2021). Due to limited survey space we do not have an equivalent measure for Brexit identities. The list of items for partisans is as below.

When [out-group party] does well in opinion polls, my day is ruined

When people criticize [out-group party], it makes me feel good

I do not have much in common with [out-group party] supporters

When I meet someone who supports [out-group party], I feel disconnected

I get angry when people praise [out-group party]

The response options were ‘strongly disagree’ to ‘strongly agree’ which are scored 1–5 and then averaged. The alpha score is 0.88 for Conservatives and 0.86 for Labour supporters and descriptive statistics and factor analyses are in Online Appendix 2. Our second measure of out-group animosity uses the stereotype questions to measure negative perceptions of the other side. Alpha scores vary from 0.67 (Conservative partisans’ views of Labour partisans) to 0.80 (Remainers’ views of Leavers).

### Measuring Personality

Our core personality measure is narcissism. We use the 18-item Narcissistic Admiration and Rivalry Questionnaire (NARQ). It is important to distinguish between self-esteem, characterized by a feeling of satisfaction with oneself, and narcissism, which is an excessive self-evaluation characterized by a sense of superiority and a desire for admiration by others (Brummelman et al., 2016; Cichocka et al., 2023; Marchlewska et al., 2019).^8^ This battery captures the latter and is well validated, with strong over time correlation and high self-other agreement (Back et al., 2013; Grosz et al., 2019; Leckelt et al., 2018). As discussed, the measure breaks down into two aspects of admiration and rivalry. For example, one of the items to measure admiration is ‘I am great’, whereas one of the items measuring rivalry is ‘I want my rivals to fail’. The 18 items use a 1–6 scale with 1 labelled ‘not agree at all’ and 6 labelled ‘agree completely’: full question wordings are in Online Appendix 3. We created two 1–6 scales, one for admiration (9 items summed and divided by 9) and one for rivalry (9 items summed and divided by 9). Alpha scores are very high, although artificially inflated due to the consistent direction of the question wordings, at 0.85 for admiration and 0.84 for rivalry. The general measure of narcissism has an alpha score of 0.87. The overall mean is 2.35, and the distribution is approximately normal with a standard deviation of 0.71.^9^ 95% of people thus score between 1 and 3.67.

We also use a 50-item version of the Big Five Aspect Scale (DeYoung et al., 2007) to control for the Big Five traits.^10^ Ten items measure each of the Big Five which avoids known problems with shorter batteries (Bakker & Lelkes, 2018). Each item is a statement to which people are asked to score themselves on a 1–5 scale from strongly disagree to strongly agree. Full details are in Online Appendix 4.

### Measuring Social Characteristics and Political Values

The final set of measures are other control variables. In the main text we just show models with narcissism and the Big Five as independent variables, but in the appendices we also present models which contain a long list of social characteristics and, where appropriate, measures of political values or type of identity. As the tables in the appendices show, the inclusion of these extra controls has little impact on the statistical significance, nor size, of the narcissism coefficients. The social characteristics are: age, gender, education, race, occupational social class, household income, trade union membership, housing tenure and religiosity. Full details are in Online Appendix 5. Political values are measured using four scales based on a battery of 24 items with 1–5 response categories from strongly disagree to strongly agree. The first two scales largely replicate previously validated measures (Evans et al., 1996) of the two main dimensions of political ideology: economic left–right and social conservative-liberal. We also include two other measures of political values, one a measure of national pride based on Heath et al. (1999) and one a measure of support for the EU. Full details are in Online Online Appendix 6.

## Analysis

**Fig. 1:**
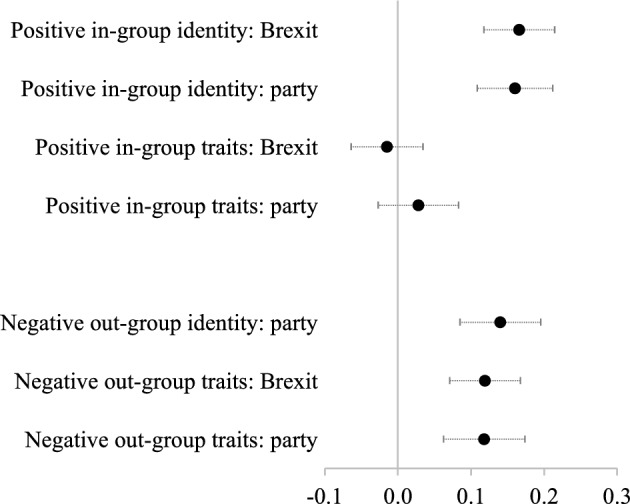
Marginal effects of narcissism on positive in-group identities, positive perceptions of in-group traits, negative out-group partisan identity and negative perceptions of out-group traits. Note: models include controls for the Big Five traits (openness to experience, conscientiousness, extraversion, agreeableness and neuroticism) and show standardized coefficients. See Online Appendix 7

In this section, we present a series of models which predict different types of affective polarization. We hypothesize that narcissism will correlate with all forms of affective polarization, but most especially out-group animosity. Figure 1 shows the results of regression models predicting people’s positive identity; positive stereotypes of the in-group; negative identity; and negative stereotypes of the out-group (see Tables A7a and A7b in Online Appendix 7 for the full models: also included in these tables are additional models with demographic and identity type controls). The coefficients are standardized, so represent the proportion of a standard deviation for each scale that is associated with a one standard deviation change in narcissism. All models include controls for the Big Five traits.

Narcissism is clearly correlated with stronger in-group affinity, stronger out-group animosity and more negative stereotypes of the out-group. The one area in which narcissism plays little role is in positive stereotyping of the in-group. This hints at the way that narcissism, with its focus on self-importance, may not necessarily translate to positive in-group perceptions since it simply inflates perceptions of the self. Nonetheless, overall we find strong support for H1 and some support for H2 in that positive stereotyping of the in-group appears unrelated to narcissism.^11^ These effects are also relatively large even though the other Big Five traits are included in the models. For example, someone high in narcissism (a standard deviation above the mean) is predicted to score 0.33 and 0.32 standard deviations more on the positive Brexit and party identity measures than someone who is low in narcissism (one standard deviation below the mean).

It is interesting to compare the size of these effects to those of the Big Five. As the models in Online Appendix 7 show, narcissism is consistently more strongly correlated with affective polarization compared to any of the Big Five traits. Probably the most consistent finding with regard to the Big Five traits is that higher levels of neuroticism predict both greater levels of in-group affinity and out-group animosity, but the standardized coefficient for neuroticism in these models tends to be smaller, and in the cases of positive in-group identity a lot smaller, than the standardized coefficient for narcissism.^12^

**Fig. 2:**
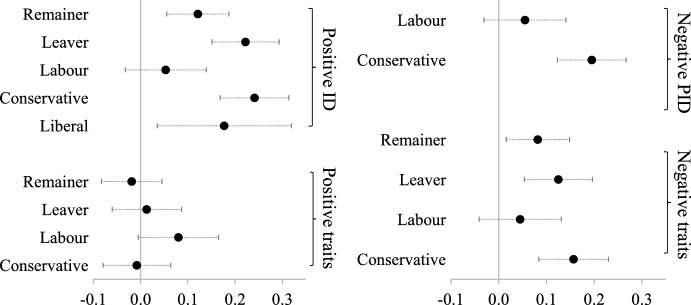
Marginal effects of narcissism on affective polarization by specific identity. Note: models include controls for the Big Five traits (openness to experience, conscientiousness, extraversion, agreeableness and neuroticism) and show standardized coefficients. See Online Appendix 9

Figure 2 breaks down the models down by identity type (see Online Appendix 9 for the full models and further models with demographic and ideological controls). The left-hand panel of Fig. 2 shows the standardized coefficients of narcissism on positive in-group affinity and positive stereotypes about the in-group, while the right-hand panel of Fig. 2 shows how narcissism is associated with negative partisanship and negative stereotypes about the out-group. The direction and size of the narcissism coefficients are fairly consistent across the different identities: narcissism is positively associated with greater positive in-group affinity, greater negative out-group animosity and greater negative out-group stereotyping for all identities. The one partial exception are Labour partisans who have a weaker, and not statistically significant, relationship between narcissism and the measures of positive and negative partisanship than Conservative and Liberal partisans. Nonetheless, overall these findings suggest that some people are simply more predisposed to affective polarization regardless of which type of political identity we look at and regardless of what side of the argument that person is on.

**Fig. 3:**
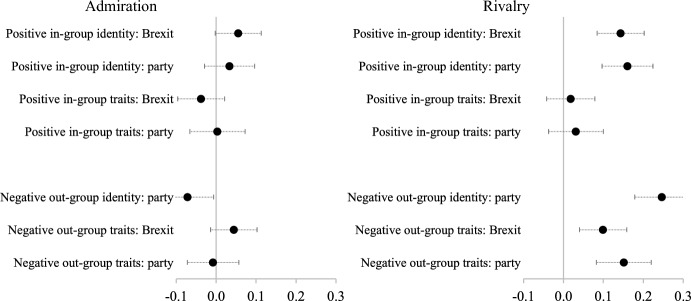
Marginal effects of the two aspects of narcissism on affective polarization. Note: models include controls for the Big Five traits (openness to experience, conscientiousness, extraversion, agreeableness and neuroticism) and show standardized coefficients. See Online Appendix 10

What of the different aspects of narcissism? We hypothesized that while both rivalry and admiration are associated with affective polarization, rivalry has the stronger association (H3). In Fig. 3 we replicate Fig. 1, but using models which simultaneously include both admiration and rivalry as predictors (see Online Appendix 10 for the full models). The left-hand panel of Fig. 3 shows the effects of admiration, the degree to which someone thinks themselves superior and more important, and the right-hand panel of Fig. 3 shows the effects of rivalry, the degree to which someone is antagonistically entitled. Holding constant rivalry, we can see that the relationship of admiration with affective polarization is very limited, and while there may be some weak association with positive in-group affinity there is also a weak association in the opposite direction with negative partisanship. On the other hand, people who score higher on rivalry consistently score higher on the in-group identity affinity scale, negative partisanship and the negative out-group perceptions scale.

The strong association between rivalry and affective polarization is clear. Those who cleave more strongly to their political in-group and express more animosity and prejudice towards their political out-group are clearly more likely to be people who score highly on this entitlement aspect of narcissism.^13^ These effects are large. If we compare rivalry to the Big Five traits, it has over twice the effect of neuroticism on positive in-group identity and negative out-identity and around one and half times the effect of neuroticism on negative out-group trait perceptions.

## Conclusion

There is considerable evidence showing that people at different ends of the ideological spectrum and with different overlapping social characteristics are more affectively polarized. In this paper, we asked whether the affectively polarized on different sides of the partisan divide may also have something in common: their personality. We show that to some extent this is the case. People high in narcissism are more affectively polarized. The more narcissistic the person, the greater their in-group attachment, out-group animosity and out-group prejudice. This suggests that there are certain similarities between people who are affectively polarized.

Our findings also highlight the importance of separating out the two elements of affective polarization when looking at possible causes. Narcissism appears somewhat more important for out-group animosity than in-group affinity. There is no effect of narcissism on positive perceptions of one’s own in-group, just on negative perceptions of the out-group. Affective polarization is generated by liking the in-group and disliking the out-group, but that does not mean that causes of affective polarization affect each aspect equally. Equally important is breaking down narcissism into its two aspects. We see that admiration, with its emphasis on self-importance and superiority, is, at most, weakly associated with a positive in-group identity. On the other hand, rivalry, with its emphasis on entitlement, and thus antagonistic self-defense, plays a role in both positive in-group identities and negative out-group identities.

One obvious implication that future research could further investigate is how different political contexts may matter for these relationships. For example, in a preliminary experimental test (see Online Appendix 11 for the full details and results), we find that an experimentally manipulated context has different effects on the impact of rivalry and admiration on affective polarization. To change this context, we asked subjects to list either positive words about their own party or negative words about the other party. In a context in which people were more focused on being cheerleaders for their own side, rather than critics of the other side, we see greater effects of admiration on in-group affinity. Self-promotion appears to spill-over into group admiration. The reverse also appears true: in situations in which people are more focused on the negative aspects of the out-group, it is rivalry, not admiration, which predicts in-group affinity. These results suggest that getting to grips with the nuances of how context interacts with personality to produce affective polarization is potentially an important next step for affective polarization researchers.

Leaving aside context, however, if we assume that narcissism is generally related to affective polarization, then these intrinsic personality similarities among the affectively polarized also have broader implications for how we understand both mass and elite affective polarization. At the mass level, there has been widespread debate about whether narcissism is increasing via generational replacement with newer cohorts argued to be more narcissistic due to changing child rearing methods and an increased emphasis on ‘self-esteem’ (see Twenge & Foster, 2010; Twenge et al., 2008; Westerman et al., 2012; but against this, see Donnellan et al., 2009; Trzesniewski et al., 2008). While the evidence for this trend remains far from conclusive, it is possible that any increases in narcissism over time could be contributing to increasing rates of mass affective polarization. At the elite level, our findings could also provide part of the explanation for why elites are typically more affectively polarized than the public. Competing for electoral office is attractive to certain types of people: those high in extraversion (Dynes et al., 2019), low in empathy (Clifford et al., 2021) and, crucially, high in narcissism (Blais & Pruysers, 2017; Hart et al., 2022; Peterson & Palmer, 2022; Watts et al., 2013). This greater narcissism makes political elites, even aside from their greater ideological polarization, potentially more intrinsically prone to affective polarization. It is also worth considering a wider notion of elite opinion formers beyond simply politicians. The views of journalists, business leaders and actors tend to be given greater precedence in public discourse, not least in the age of social media, yet these are precisely the professions which tend to attract people who are more narcissistic (Kowalski et al., 2017; Rothman & Lichter, 1985) and who are thus, on average, more likely to be affectively polarized. If political elites, or this wider elite, do cue voters then mass affective polarization is being partially generated by the fact that narcissists are more likely to enter these elite professions. Our findings thus help us further understand the phenomenon of affective polarization from both above and below.

Nonetheless, there are, as always, caveats to our findings. Perhaps most importantly, and as discussed earlier, our findings can only show a correlation, not a causal relationship. While most personality research assumes that personality traits are fixed from early childhood and are thus causes of any political attitudes and behavior, it is increasingly clear that any relationship between political views and personality may be reciprocal. For example, using panel and experimental data, Bakker et al. (2021) show that there is a weak effect of political attitudes on the Big Five personality traits. Similarly Boston et al. (2018) find that the Big Five, especially openness to experience, are predicted by political variables such as presidential approval. These important findings should make us cautious about not over interpreting our results in a strictly causal manner.

Indeed, there is an argument that strong group identification could produce narcissism. Certainly there is evidence that children who are told they are superior become more narcissistic and there is an analogy there with being in a (political) group which tells each other they are superior (Brummelman et al., 2015). Nonetheless, this evidence for change is from early years’ socialization, not adult life. It is also the case that the mechanisms generally suggested for a causal path from politics to personality seem less likely to produce a causal relationship from polarization to personality than from ideology to personality. We show that strong identifiers on both sides are similar. This does not suggest a process whereby people view their personality traits through a prism of their political outlook, nor that people necessarily adopt the traits of people similar to them politically. Equally, our data are partially helpful in resolving worries about causal direction. We take the advice of Boston et al. (2018, p. 862) and employ ‘a more comprehensive battery than TIPI for the main trait of interest’. Our measurement of personality is not the ten item standard battery for the Big Five, but rather 18 items that separately measure narcissism and then 50 items that measure the Big Five. Equally, our personality questions are measured three months earlier than the political questions, so we might expect that ‘political salience in the survey context’ (Bakker et al., 2021, p. 32) is less of an issue for our study.

That is not to say that we can rule out a reciprocal relationship between narcissism, or personality more generally, and polarization, but it seems likely that even if polarization causes narcissism, narcissism also causes polarization. If so, the challenges for a less polarized polity may be two-fold. First, at the mass level there is the matter of engaging not just more narcissistic people intrinsically attracted to political strife, but also interesting those who are more reluctant to join political groups. Second, at the elite level, there is the difficulty of recruiting less narcissistic people who may be less intrinsically enthusiastic about a political career. Neither challenge has obvious solutions. Nonetheless, by enhancing our understanding of the kind of people who are more likely to become affectively polarized, we may be able to develop more effective interventions to reduce the potentially harmful effects of such polarization.

## Supplementary Information

Below is the link to the electronic supplementary material.

Supplementary file1 (DOCX 89 KB)

## Data Availability

The data and replication files are available on the Harvard dataverse: https://doi.org/10.7910/DVN/PUYD5U.

## Footnotes

1 The HEXACO model of personality includes a sixth trait called ‘honesty-humility’. This sixth trait correlates with all three of the Dark Triad including narcissism (Lee & Ashton, 2005) further suggesting that narcissism is a real additional facet of personality beyond the Big Five.

2 When referring to narcissism as a personality trait, we do not therefore include ‘vulnerable narcissism’. This is an important part of narcissistic personality disorders, but most concepts and measures of narcissism as a trait focus on ‘grandiose narcissism’ and do not cover narcissistic vulnerability. One exception is the Pathological Narcissism Inventory (PNI) which has its roots in clinical assessments of narcissism (Pincus et al., 2009).

3 For example, the Narcissistic Personality Inventory (NPI) is commonly used. One problem with this measure is its lack of coverage of the entitlement aspect of narcissism and over-emphasis on ‘elements of personality linked with confidence, assertiveness, and beliefs of leadership potential’ (Ackerman et al., 2011, p82).

4 The other Big Five trait which is generally found to be important is openness to experience. Satherley et al (2020) show, using a large sample from New Zealand, that people lower in openness to experience are generally less hostile towards the outgroup party.

5 In our data, agreeableness is correlated at -.37 with narcissism. The next highest correlation between narcissism and one of the Big Five traits is for extraversion (.17).

6 We have a more limited set of dependent variables simultaneously measured in Wave 1. These show very similar relationships between narcissism and affective polarisation suggesting that any political changes between March and July 2021 did not alter the effects. See Table A7c in Online Appendix 7.

7 This measure is based on the classic Civic Culture study in which respondents were asked to think about ‘what sorts of people support and vote for the different parties’ and then indicate whether a set of positive and negative terms applied to party supporters (Almond & Verba, 1963). This included positive traits such as ‘intelligent’ and ‘interested in the welfare of humanity’ and negative traits such as ‘selfish’ and ‘ignorant’. Similar measures of the perceived traits of fellow, and opposing, partisans have been used in the literature as a measure of affective polarization (Iyengar et al., 2012; West & Iyengar, 2022; Busby et al. 2021). Perceptions of the traits of two sides are interesting because they reveal whether there is prejudice as well as animosity towards the other side. In fact, this type of partisan prejudice in the US is often compared to racial prejudice and stereotyping (Iyengar & Westwood, 2015).

8 Nonetheless, one limitation of our study is the lack of an equivalent measure of self-esteem. This means that we cannot measure the effects of narcissism holding constant self-esteem, nor compare the magnitudes of any effects of narcissism and self-esteem.

9 This is in line with previous representative and convenience samples in which means are around 2.5 (Back et al. 2013; Leckelt et al., 2018).

10 Unfortunately we do not have measures of the other two Dark Triad traits (Machiavellianism and psychopathy), so it may be that some of our analysis is potentially picking up associations between other elements of the Dark Triad and affective polarization.

11 The difference between the size of the narcissism coefficient on positive partisan traits and negative partisan traits is statistically significant and the difference between the narcissism coefficient on positive Brexit traits and negative Brexit traits is also statistically significant, but as the figure suggests there is no difference in the size of the narcissism coefficient when predicting positive partisanship compared to negative partisanship. Appendix 8 presents seemingly unrelated regression models showing these tests of coefficient equalities.

12 Since neuroticism is, at least partially, a measure of how strongly people feel negative emotions it seems sensible that people with a more pervasive sense of uncertainty and threat will cleave more strongly to in-groups and be more hostile to out-groups. This also fits with work by Chen and Palmer (2018) who show that people high in neuroticism tend to engage in greater out-group stereotyping. In terms of the other Big Five traits, there are also some more inconsistent positive associations between affective polarisation and both agreeableness and openness to experience.

13 Tests of differences between the standardized coefficients show that rivalry is statistically significantly more strongly associated with positive Brexit identity, positive party identity, negative party identity and negative party traits at the 5% level. The different effects of rivalry and admiration on all measures of affective polarization are consistent for both Remainers and Leavers. However, unsurprisingly, the weaker association between general narcissism and affective polarisation for Labour supporters is seen in a weaker association between rivalry and polarization for Labour supporters compared to both Conservative and Liberal Democrat supporters.

## Declarations

### Pre-registration

Our study was not pre-registered.
