# Supplementary material for: Narcissism and Affective Polarization
Source: Polit Behav. 2024 Aug 2;47(2):599–618. doi: 10.1007/s11109-024-09963-5 (PMC12053521; doi:10.1007/s11109-024-09963-5)
Supplement: Supplementary file 1 — Supplementary file1 (DOCX 89 KB) [file 11109_2024_9963_MOESM1_ESM.docx]

**Narcissism and Affective Polarization**

**ONLINE APPENDICES**

**Table of Contents:**

Appendix 1 – Political identities

Appendix 2 – Descriptive statistics for measures of positive and negative political identity

Appendix 3 – Narcissistic Admiration and Rivalry Questionnaire

Appendix 4 – Big Five Aspect Scale questionnaire

Appendix 5 – Social characteristics

Appendix 6 – Political ideology questionnaire

Appendix 7 – Regression tables for Figure 1

Appendix 8 – Regression tables for footnote 12

Appendix 9 – Regression tables for Figure 2

Appendix 10 – Regression tables for Figure 3

Appendix 11 – Details of context experiment

**Appendix 1 – Political identities**

**Party identity**

*Generally speaking, do you think of yourself as Labour, Conservative, Liberal Democrat or what?*

[IF NO] *Do you generally think of yourself as a little closer to one of the parties than the others?*

*Party identities (percentage of retained wave 1 sample at wave 2)*

| Conservative | 35% |
| --- | --- |
| Labour | 24% |
| Liberal Democrat | 9% |
| Green | 5% |
| Scottish National Party | 3% |
| Other | 2% |
| None or DK | 21% |
|  |  |
| N | 2718 |

**Brexit identity**

*Since the EU referendum, some people now think of themselves as Leavers and Remainers, do you think of yourself as a Leaver, a Remainer, or neither a Leaver or Remainer?*

[IF NO] *Do you generally think of yourself as a little closer to Remainers or Leavers, or do you not feel close to either?’*

*Brexit identities (percentage of retained wave 1 sample at wave 2)*

| Remainer | 41% |
| --- | --- |
| Leaver | 35% |
| Neither or DK | 24% |
|  |  |
| N | 2719 |

**Party and Brexit identities**

*Party and Brexit identities (percentage of retained wave 1 sample at wave 2)*

|  |  | Brexit identity |  |  |
| --- | --- | --- | --- | --- |
|  |  | Remainer | Leaver | Neither |
| Party identity | Conservative | 6% | 22% | 7% |
|  | Labour | 16% | 4% | 4% |
|  | Liberal Democrat | 7% | 1% | 1% |
|  | Green | 4% | 1% | 1% |
|  | Scottish National Party | 3% | - | 1% |
|  | Other | - | 1% | - |
|  | None or DK | 6% | 5% | 10% |

Note: Total N = 2718.

**Appendix 2 – Measures of positive and negative political identity**

*Descriptive statistics – positive and negative political identity*

| *Measure* | *Mean* | *Standard deviation* | *N* |
| --- | --- | --- | --- |
|  |  |  |  |
| Positive in-group party identity | 2.85 | .73 | 1850 |
| Positive in-group Brexit identity | 3.18 | .78 | 2060 |
|  |  |  |  |
| Positive in-group partisan stereotypes | 3.56 | .61 | 1611 |
| Positive in-group Brexit stereotypes | 3.74 | .71 | 2060 |
|  |  |  |  |
| Negative out-group party identity | 2.99 | .84 | 1611 |
|  |  |  |  |
| Negative out-group partisan stereotypes | 3.45 | .77 | 1611 |
| Negative out-group Brexit stereotypes | 3.41 | .85 | 2060 |
|  |  |  |  |

Note: Retained wave 1 sample at wave 2.

*Factor loadings - positive in-group identity and negative out-group identity*

|  | *Conservative* | *Labour* | *Remainer* | *Leaver* |
| --- | --- | --- | --- | --- |
| In-group positivity |  |  |  |  |
| *I usually say ‘we’ instead of ‘they’* | .70 | .68 | .68 | .69 |
| *Criticism is a personal insult* | .78 | .72 | .72 | .71 |
| *I have a lot in common* | .64 | .65 | .69 | .77 |
| *I feel connected* | .84 | .76 | .85 | .85 |
| *Praise makes me feel good* | .83 | .78 | .86 | .85 |
|  |  |  |  |  |
| Single factor % variance | 65.9 | 61.3 | 65.9 | 67.7 |
|  |  |  |  |  |
| Out-group negativity |  |  |  |  |
| *Does well, my day is ruined* | .77 | .66 |  |  |
| *Criticism makes me feel good* | .63 | .66 |  |  |
| *I do not have much in common* | .82 | .82 |  |  |
| *I feel disconnected* | .81 | .81 |  |  |
| *Praise makes me angry* | .79 | .77 |  |  |
|  |  |  |  |  |
| Single factor % variance | 66.8 | 64.3 |  |  |
|  |  |  |  |  |

Note: Retained wave 1 sample at wave 2. See main text for full question wordings. Factor analysis uses maximum likelihood extraction method. All scales have one-factor solutions with second factors below a 0.7 Eigenvalue.

**Appendix 3 – Narcissistic Admiration and Rivalry Questionnaire**

‘Please indicate how much the following statements apply to you from ‘not agree at all’ to ‘agree completely’. Please be as honest as possible. Rely on your initial feeling and do not think too much about each statement.’

I am great

I will someday be famous

I deserve to be seen as a great personality

I show others how special I am

I enjoy my successes very much

Being a special person gives me a lot of strength

Most of the time I am able to draw people's attention to myself in conversations

I manage to be the centre of attention with my outstanding contributions

Mostly, I am very adept with dealing with people

Most people won't achieve anything

Other people are worth nothing

Most people are somehow losers

I secretly take pleasure in the failure of my rivals

I want my rivals to fail

I enjoy it when another person is inferior to me

I become annoyed if another person steals the show from me

I often get annoyed when I am criticised

I can barely stand it if another person is at the centre of events

The order of all 18 statements is randomized for respondents. There are six response categories with 1 labelled ‘not agree at all’ and 6 labelled ‘agree completely’. The first 9 statements measure the admiration sub-trait and the last 9 measure the rivalry sub-trait.

*Descriptive statistics – Narcissism and aspects*

| *Trait* | *Mean* | *Standard deviation* | *Cronbach’s alpha* |
| --- | --- | --- | --- |
|  |  |  |  |
| Overall narcissism scale | 2.35 | .71 | .87 |
|  |  |  |  |
| Admiration | 2.55 | .83 | .85 |
| Rivalry | 2.15 | .83 | .84 |
|  |  |  |  |
| Correlation between admiration and rivalry | 0.43 |  |  |
|  |  |  |  |

Note: Wave 1 sample of 3,552 respondents

**Appendix 4 – Big Five Aspect Scale questionnaire**

‘Here are a number of characteristics that may, or may not, describe you. Please indicate how much you agree with each statement listed below. Please be as honest as possible. Rely on your initial feeling and do not think too much about each statement.’

I get angry easily

I rarely get irritated

I get upset easily

I am not easily annoyed

I keep my emotions under control

I feel comfortable with myself

I feel threatened easily

I rarely feel depressed

I worry about things

I am easily discouraged

I can’t be bothered with other’s needs

I sympathize with others’ feelings

I take an interest in other people’s lives

I feel others’ emotions

I am not interested in other people’s problems

I insult people

I avoid imposing my will on others

I rarely put people under pressure

I take advantage of others

I love a good fight

I carry out my plans

I waste my time

I find it difficult to get down to work

I mess things up

I finish what I start

I leave my belongings around

I like order

I keep things tidy

I want everything to be "just right"

I dislike routine

I keep others at a distance

I warm up quickly to others

I am not a very enthusiastic person

I have a lot of fun

I make friends easily

I see myself as a good leader

I do not have an assertive personality

I take charge

I wait for others to lead the way

I hold back my opinions

I am quick to understand things

I have difficulty understanding abstract ideas

I avoid philosophical discussions

I formulate ideas clearly

I learn things slowly

I believe in the importance of art

I get deeply immersed in music

I do not like poetry

I see beauty in things that others might not notice

I seldom notice the emotional aspects of paintings and pictures

The order of all 50 statements is randomized for respondents. The response categories are: strongly disagree, disagree, neither agree nor disagree, agree, strongly agree. The first 10 statements measure neuroticism, the next 10 measure agreeableness, the next 10 measure conscientiousness, the next 10 measure extraversion and the final 10 measure openness to experience.

*Descriptive statistics – Big Five traits*

| *Trait* | *Mean* | *Standard deviation* | *Cronbach’s alpha* |
| --- | --- | --- | --- |
|  |  |  |  |
| Neuroticism | 2.92 | .68 | .85 |
| Agreeableness | 3.78 | .52 | .78 |
| Conscientiousness | 3.50 | .56 | .78 |
| Extraversion | 3.10 | .57 | .80 |
| Openness to experience | 3.31 | .54 | .73 |
|  |  |  |  |

Note: Wave 1 sample of 3,552 respondents

**Appendix 5 – Social characteristics**

*Social characteristic measures*

| *Social characteristic* | *Details* |
| --- | --- |
|  |  |
| Age | Age in years |
|  |  |
| Gender | Man; woman. |
|  |  |
| Race | White British; other white; non-white; other. |
|  |  |
| Education | No qualifications; apprenticeship; GCSEs; A-Levels; degree; higher degree. |
|  |  |
| Occupational social class | Old middle class; new middle class; junior middle class; personal service; own account workers; foremen; working class; unknown (based on Evans and Tilley 2017). |
|  |  |
| Household income | Quintiles. |
|  |  |
| Trade union membership | Current member; previously member; never member; unknown. |
|  |  |
| Housing tenure | Owner; owner with mortgage; private renter; public renter; other. |
|  |  |
| Religious attendance | Weekly or fortnightly; monthly or bimonthly; once a year or less; never. |
|  |  |

**Appendix 6 – Political ideology questionnaire**

‘How much do you agree or disagree with the following statements’:

Government should redistribute income from the better off to those who are less well off

Big business benefits owners at the expense of workers

Ordinary working people do not get their fair share of the nation’s wealth

There is one law for the rich and one for the poor

Management will always try to get the better of employees if it gets the chance

Strong trade unions protect employees’ working conditions and wages

Major public services and industries ought to be in state ownership

Young people today don’t have enough respect for traditional British values

For some crimes, the death penalty is the most appropriate sentence

Schools should teach children to obey authority

Censorship of films and magazines is necessary to uphold moral standards

People who break the law should be given stiffer sentences

The amount of immigration to Britain should be decreased

Gay couples should not be allowed to get married

European courts should be able to make decisions about human rights cases in Britain

Some laws are better made at the European level

The British Parliament should not be able to override all EU laws

Britain should hold another referendum on re-joining the EU

Britain loses out by not being a member of the EU

I feel proud to be British

Being a member of the EU undermined Britain’s distinctive identity

I would rather be a citizen of Britain than of any other country in the world

People in Britain are too ready to criticise their country

The world would be a better place if people from other countries were more like the British

The order of all 24 statements is randomized for respondents. The response categories are: strongly disagree, disagree, neither agree nor disagree, agree, strongly agree. The first 7 statements measure economic left-right position, the next 7 measure social liberal-conservative position, the next 5 measure EU integration position and the final 5 measure national pride.

*Descriptive statistics – Political attitude scales*

| *Trait* | *Mean* | *Standard deviation* | *Cronbach’s alpha* |
| --- | --- | --- | --- |
|  |  |  |  |
| Economic left-right | 3.64 | .70 | .83 |
| Social liberal-conservative | 3.26 | .80 | .80 |
| EU integration | 2.75 | 1.14 | .91 |
| National pride | 3.26 | .87 | .82 |
|  |  |  |  |

Note: Retained wave 1 sample at wave 2 of 2,719 respondents

**Appendix 7 – Regression tables for Figure 1**

Table A7a: *OLS models predicting positive in-group feeling*

|  | *Positive in-group identity* | | | | | | *Positive in-group traits* | | | | | |
| --- | --- | --- | --- | --- | --- | --- | --- | --- | --- | --- | --- | --- |
|  | *Brexit identity* | | | *Party identity* | | | *Brexit identity* | | | *Party identity* | | |
|  |  |  |  |  |  |  |  |  |  |  |  |  |
| *Narcissism* | .16* | .19* | .19* | .16* | .18* | .19* | -.01 | .01 | .01 | .03 | .05 | .05 |
|  |  |  |  |  |  |  |  |  |  |  |  |  |
| *Neuroticism* | .06* | .09* | .09* | .06* | .09* | .09* | -.03 | -.00 | -.00 | -.03 | -.03 | -.03 |
| *Agreeableness* | .09* | .10* | .09* | .05 | .06* | .05 | .11* | .12* | .13* | .14* | .14* | .13* |
| *Conscientiousness* | -.01 | -.01 | -.00 | .01 | .01 | .01 | .02 | .02 | .01 | .05 | .05 | .05 |
| *Extraversion* | .03 | .02 | .02 | .05 | .03 | .03 | .04 | .03 | .03 | .05 | .04 | .04 |
| *Open to experience* | .03 | .05* | .05* | .05 | .09* | .08* | .08* | .09* | .10* | .07* | .08* | .07* |
|  |  |  |  |  |  |  |  |  |  |  |  |  |
| *Demog controls* |  | X | X |  | X | X |  | X | X |  | X | X |
| *Identity controls* |  |  | X |  |  | X |  |  | X |  |  | X |
|  |  |  |  |  |  |  |  |  |  |  |  |  |
| *Adjusted R^2^* | .03 | .05 | .05 | .03 | .07 | .07 | .03 | .04 | .04 | .04 | .04 | .04 |
| *N* | 1986 | 1986 | 1986 | 1748 | 1748 | 1748 | 1986 | 1986 | 1986 | 1515 | 1515 | 1515 |

Note: * p<0.05. Standardized coefficients. Demographic controls: sex; educational qualifications; race; occupational class; household income quintiles; union membership; housing tenure and religiosity. Identity controls: Remainer/ Leaver for Brexit identity and Conservative/ Labour/ Liberal Democrat for party identity.

Table A7b: *OLS models predicting negative out-group feeling*

|  | *Negative out-group identity* | | | *Negative out-group traits* | | | | | |
| --- | --- | --- | --- | --- | --- | --- | --- | --- | --- |
|  | *Party identity* | | | *Brexit identity* | | | *Party identity* | | |
|  |  |  |  |  |  |  |  |  |  |
| *Narcissism* | .14* | .14* | .15* | .12* | .07* | .07* | .12* | .08* | .08* |
|  |  |  |  |  |  |  |  |  |  |
| *Neuroticism* | .09* | .12* | .11* | .06* | .07* | .07* | .10* | .09* | .08* |
| *Agreeableness* | -.02 | -.00 | -.03 | -.04 | -.00 | -.02 | -.03 | -.00 | -.03 |
| *Conscientiousness* | -.07* | -.06* | -.04 | -.01 | -.01 | .01 | -.04 | -.04 | -.02 |
| *Extraversion* | -.05 | -.05 | -.04 | -.04 | -.02 | -.01 | -.04 | -.00 | .00 |
| *Open to experience* | .06* | .07* | .03 | .07* | .02 | .00 | .10* | .06* | .03 |
|  |  |  |  |  |  |  |  |  |  |
| *Demog controls* |  | X | X |  | X | X |  | X | X |
| *Identity controls* |  |  | X |  |  | X |  |  | X |
|  |  |  |  |  |  |  |  |  |  |
| *Adjusted R^2^* | .04 | .07 | .11 | .03 | .06 | .08 | .04 | .08 | .12 |
| *N* | 1515 | 1515 | 1515 | 1986 | 1986 | 1986 | 1515 | 1515 | 1515 |

Note: * p<0.05. Standardized coefficients. Demographic controls: sex; educational qualifications; race; occupational class; household income quintiles; union membership; housing tenure and religiosity. Identity controls: Remainer/ Leaver for Brexit identity and Conservative/ Labour/ Liberal Democrat for party identity.

Table A7c: *OLS models predicting affective polarization (both independent and dependent variables measured in wave 1)*

|  | *Positive identity* | | | | *Positive in-group traits* | | | | *Negative partisanship* | | *Negative out-group traits* | | | |
| --- | --- | --- | --- | --- | --- | --- | --- | --- | --- | --- | --- | --- | --- | --- |
|  | *Brexit* | | *Party* | | *Brexit* | | *Party* | |  | | *Brexit* | | *Party* | |
|  |  |  |  |  |  |  |  |  | Not measured in wave 1 | |  |  |  |  |
| *Narcissism* | .15* | .19* | .15* | .19* | -.02 | .03 | .02 | .03 |  |  | .14* | .11* | .07* | .03 |
|  |  |  |  |  |  |  |  |  |  |  |  |  |  |  |
| *Neuroticism* | .10* | .13* | .09* | .11* | -.07* | -.05* | -.03 | -.02 |  |  | .02 | .03 | .09* | .08* |
| *Agreeableness* | .04 | .04 | .02 | .02 | .05* | .09* | .11* | .12* |  |  | -.08* | -.07* | -.02 | -.02 |
| *Conscientiousness* | -.00 | -.01 | .02 | .02 | .06* | .03 | .05 | .04 |  |  | -.06* | -.04* | -.10* | -.05* |
| *Extraversion* | .07* | .05* | .04 | .02 | .03 | .00 | .01 | .00 |  |  | -.07* | -.04 | -.02 | .02 |
| *Open to experience* | .05* | .08* | .03 | .07* | -.02 | .06 | .00 | .03 |  |  | .07* | .03 | .07* | .02 |
|  |  |  |  |  |  |  |  |  |  |  |  |  |  |  |
| *Demog controls* |  | X |  | X |  | X |  | X |  |  |  | X |  | X |
| *Identity controls* |  | X |  | X |  | X |  | X |  |  |  | X |  | X |
|  |  |  |  |  |  |  |  |  |  |  |  |  |  |  |
| *Adjusted R^2^* | .04 | .10 | .03 | .06 | .02 | .09 | .02 | .02 |  |  | .04 | .09 | .03 | .10 |
| *N* | 2303 | 2303 | 2027 | 2027 | 2798 | 2798 | 1986 | 1986 |  |  | 2798 | 2798 | 1986 | 1986 |

Note: * p<0.05. Standardized coefficients. All variables measured at Wave 1. Demographic controls: sex; educational qualifications; race; occupational class; household income quintiles; union membership; housing tenure and religiosity. Identity controls: Remainer/ Leaver for Brexit identity and Conservative/ Labour/ Liberal Democrat for party identity.

**Appendix 8 – Regression tables for footnote 10**

Table A8: *Seemingly unrelated regression models predicting affective polarization*

|  | *Partisanship* | | *Partisan traits* | | *Brexit traits* | |
| --- | --- | --- | --- | --- | --- | --- |
|  | *Positive* | *Negative* | *Positive* | *Negative* | *Positive* | *Negative* |
| *Narcissism coefficient* | .17* | .17* | -.02 | .14* | .03 | .13* |
|  |  |  |  |  |  |  |
| *Coefficient difference* |  | -.00 |  | -.16* |  | -.11* |

Note: * p<0.05. Models also include controls for the Big Five traits.

**Appendix 9 – Regression tables for Figure 2**

Table A9a: *OLS models predicting positive identity*

|  | Leavers | | | Remainers | | | Conservatives | | | Labour | | | Liberal Democrats | | |
| --- | --- | --- | --- | --- | --- | --- | --- | --- | --- | --- | --- | --- | --- | --- | --- |
|  |  |  |  |  |  |  |  |  |  |  |  |  |  |  |  |
| *Narcissism* | .22* | .25* | .22* | .12* | .14* | .14* | .24* | .27* | .20* | .05 | .08 | .07 | .20* | .21* | .19* |
|  |  |  |  |  |  |  |  |  |  |  |  |  |  |  |  |
| *Neuroticism* | .01 | .05 | .05 | .11* | .14* | .10* | .07 | .09* | .09* | .05 | .09 | .08 | .08 | .07 | .06 |
| *Agreeableness* | .12* | .10* | .12* | .07* | .07* | .04 | .09* | .08* | .08* | .02 | .03 | .02 | -.04 | -.02 | -.07 |
| *Conscientiousness* | -.03 | -.01 | -.06 | .01 | .02 | .05 | .03 | .05 | .01 | -.01 | -.02 | .01 | -.05 | -.04 | -.01 |
| *Extraversion* | .05 | .05 | .03 | .02 | -.01 | -.02 | .04 | .03 | .02 | .07 | .04 | .05 | .03 | .01 | .00 |
| *Open to experience* | -.03 | .01 | .04 | .08* | .09* | .04 | .00 | .02 | .04 | .13* | .19* | .14* | -.03 | .00 | -.00 |
|  |  |  |  |  |  |  |  |  |  |  |  |  |  |  |  |
| *Demog controls* |  | X | X |  | X | X |  | X | X |  | X | X |  |  | X |
| *Values controls* |  |  | X |  |  | X |  |  | X |  |  | X |  |  | X |
|  |  |  |  |  |  |  |  |  |  |  |  |  |  |  |  |
| *Adjusted R^2^* | .04 | .08 | .19 | .03 | .05 | .16 | .05 | .09 | .21 | .02 | .04 | .09 | .04 | .12 | .22 |
| *N* | 930 | 930 | 930 | 1055 | 1055 | 1055 | 894 | 894 | 894 | 620 | 620 | 620 | 232 | 232 | 232 |

Note: * p<0.05. Standardized coefficients. Demographic controls: sex; educational qualifications; race; occupational class; household income quintiles; union membership; housing tenure and religiosity. Ideology controls: economic left-right ideology, social conservative-liberal ideology, national pride and support for EU membership.

Table A9b: *OLS models predicting perceptions of positive in-group traits*

|  | Leavers | | | Remainers | | | Conservatives | | | Labour | | |
| --- | --- | --- | --- | --- | --- | --- | --- | --- | --- | --- | --- | --- |
|  |  |  |  |  |  |  |  |  |  |  |  |  |
| *Narcissism* | .01 | .03 | .03 | -.02 | -.01 | -.01 | -.01 | .04 | .02 | .08 | .07 | .08 |
|  |  |  |  |  |  |  |  |  |  |  |  |  |
| *Neuroticism* | -.10* | -.08* | -.09* | .05 | .06 | .03 | -.05 | -.06 | -.06 | .02 | .02 | .01 |
| *Agreeableness* | .12* | .11* | .13* | .11* | .12* | .10* | .15* | .14* | .16* | .13* | .13* | .11* |
| *Conscientiousness* | .01 | .01 | -.04 | .02 | .03 | .05 | .02 | .03 | .01 | .08 | .09* | .13* |
| *Extraversion* | .04 | .03 | .03 | .03 | .02 | .02 | .07 | .08* | .06 | .01 | .02 | .05 |
| *Open to experience* | .04 | .07 | .09* | .13* | .12* | .08* | .06 | .06 | -.06 | .08 | .08 | -.00 |
|  |  |  |  |  |  |  |  |  |  |  |  |  |
| *Demog controls* |  | X | X |  | X | X |  | X | X |  | X | X |
| *Values controls* |  |  | X |  |  | X |  |  | X |  |  | X |
|  |  |  |  |  |  |  |  |  |  |  |  |  |
| *Adjusted R^2^* | .03 | .04 | .15 | .04 | .05 | .14 | .05 | .05 | .23 | .03 | .03 | .12 |
| *N* | 930 | 930 | 930 | 1055 | 1055 | 1055 | 894 | 894 | 894 | 620 | 620 | 620 |

Note: * p<0.05. Standardized coefficients. Demographic controls: sex; educational qualifications; race; occupational class; household income quintiles; union membership; housing tenure and religiosity. Ideology controls: economic left-right ideology, social conservative-liberal ideology, national pride and support for EU membership.

Table A9c: *OLS models predicting negative partisanship*

|  | Conservatives | | | Labour | | |
| --- | --- | --- | --- | --- | --- | --- |
|  |  |  |  |  |  |  |
| *Narcissism* | .20* | .24* | .21* | .06 | .05 | .07 |
|  |  |  |  |  |  |  |
| *Neuroticism* | .08* | .13* | .11* | .05 | .08 | .06 |
| *Agreeableness* | -.05 | -.04 | -.03 | -.05 | -.04 | -.05 |
| *Conscientiousness* | .01 | .03 | -.01 | -.12* | -.09* | -.02 |
| *Extraversion* | -.01 | -.01 | -.03 | -.07 | -.06 | .01 |
| *Open to experience* | -.11* | -.09* | -.07* | .17* | .17* | .03 |
|  |  |  |  |  |  |  |
| *Demog controls* |  | X | X |  | X | X |
| *Values controls* |  |  | X |  |  | X |
|  |  |  |  |  |  |  |
| *Adjusted R^2^* | .06 | .12 | .23 | .04 | .04 | .26 |
| *N* | 894 | 894 | 894 | 620 | 620 | 620 |

Note: * p<0.05. Standardized coefficients. Demographic controls: sex; educational qualifications; race; occupational class; household income quintiles; union membership; housing tenure and religiosity. Ideology controls: economic left-right ideology, social conservative-liberal ideology, national pride and support for EU membership.

Table A9d: *OLS models predicting perceptions of negative out-group traits*

|  | Leavers | | | Remainers | | | Conservatives | | | Labour | | |
| --- | --- | --- | --- | --- | --- | --- | --- | --- | --- | --- | --- | --- |
|  |  |  |  |  |  |  |  |  |  |  |  |  |
| *Narcissism* | .12* | .11* | .11* | .08* | .03 | .05 | .16* | .11* | .08* | .04 | .03 | .06 |
|  |  |  |  |  |  |  |  |  |  |  |  |  |
| *Neuroticism* | .03 | .04 | .02 | .09* | .11* | .06 | .09* | .09* | .06 | .05 | .06 | .04 |
| *Agreeableness* | -.10* | -.06 | -.05 | -.04 | -.01 | -.04 | -.09* | -.06 | -.04 | -.03 | -.01 | -.03 |
| *Conscientiousness* | .01 | .00 | -.04 | .01 | .02 | .06 | .03 | .03 | -.03 | -.07 | -.05 | .02 |
| *Extraversion* | -.04 | -.02 | -.01 | -.02 | .01 | .02 | -.01 | .04 | .02 | -.04 | -.01 | .07 |
| *Open to experience* | -.06 | -.07 | -.06 | .10* | .05 | -.02 | -.03 | -.06 | -.03 | .14* | .11* | -.02 |
|  |  |  |  |  |  |  |  |  |  |  |  |  |
| *Demog controls* |  | X | X |  | X | X |  | X | X |  | X | X |
| *Values controls* |  |  | X |  |  | X |  |  | X |  |  | X |
|  |  |  |  |  |  |  |  |  |  |  |  |  |
| *Adjusted R^2^* | .03 | .06 | .18 | .02 | .05 | .23 | .05 | .09 | .20 | .02 | .02 | .25 |
| *N* | 930 | 930 | 930 | 1055 | 1055 | 1055 | 894 | 894 | 894 | 620 | 620 | 620 |

Note: * p<0.05. Standardized coefficients. Demographic controls: sex; educational qualifications; race; occupational class; household income quintiles; union membership; housing tenure and religiosity. Ideology controls: economic left-right ideology, social conservative-liberal ideology, national pride and support for EU membership.

**Appendix 10 – Regression tables for Figure 3**

Table A10a: *OLS models predicting positive in-group feeling*

|  | *Positive in-group identity* | | | | | | *Positive in-group traits* | | | | | |
| --- | --- | --- | --- | --- | --- | --- | --- | --- | --- | --- | --- | --- |
|  | *Brexit identity* | | | *Party identity* | | | *Brexit identity* | | | *Party identity* | | |
|  |  |  |  |  |  |  |  |  |  |  |  |  |
| *Rivalry* | .14* | .14* | .14* | .16* | .15* | .16* | .02 | .02 | .02 | .03 | .04 | .04 |
| *Admiration* | .06 | .08* | .08* | .04 | .07* | .07* | -.03 | -.01 | -.00 | .01 | .02 | .02 |
|  |  |  |  |  |  |  |  |  |  |  |  |  |
| *Neuroticism* | .06* | .08* | .08* | .05 | .08* | .08* | -.03 | -.01 | -.01 | -.03 | -.03 | -.03 |
| *Agreeableness* | .10* | .10* | .10* | .07* | .07* | .06* | .12* | .12* | .13* | .15* | .14* | .14* |
| *Conscientiousness* | -.00 | -.00 | -.00 | .01 | .01 | .02 | .02 | .02 | .01 | .05 | .05 | .05 |
| *Extraversion* | .05 | .03 | .03 | .08* | .05 | .05 | .05 | .03 | .03 | .05 | .06 | .05 |
| *Open to experience* | .03 | .06* | .06* | .06* | .09* | .08* | .08* | .09* | .10* | .07* | .08* | .07* |
|  |  |  |  |  |  |  |  |  |  |  |  |  |
| *Demog controls* |  | X | X |  | X | X |  | X | X |  | X | X |
| *Identity controls* |  |  | X |  |  | X |  |  | X |  |  | X |
|  |  |  |  |  |  |  |  |  |  |  |  |  |
| *Adjusted R^2^* | .03 | .05 | .05 | .04 | .07 | .08 | .03 | .04 | .04 | .04 | .04 | .04 |
| *N* | 1986 | 1986 | 1986 | 1748 | 1748 | 1748 | 1986 | 1986 | 1986 | 1515 | 1515 | 1515 |

Note: * p<0.05. Standardized coefficients. Demographic controls: sex; educational qualifications; race; occupational class; household income quintiles; union membership; housing tenure and religiosity. Identity controls: Remainer/ Leaver for Brexit identity and Conservative/ Labour/ Liberal Democrat for party identity.

Table A10b: *OLS models predicting negative out-group feeling*

|  | *Negative out-group identity* | | | *Negative out-group traits* | | | | | |
| --- | --- | --- | --- | --- | --- | --- | --- | --- | --- |
|  | *Party identity* | | | *Brexit identity* | | | *Party identity* | | |
|  |  |  |  |  |  |  |  |  |  |
| *Rivalry* | .24* | .22* | .23* | .10* | .08* | .08* | .15* | .13* | .14* |
| *Admiration* | -.07 | -.05 | -.05 | .05 | .01 | -.01 | -.01 | -.04 | -.05 |
|  |  |  |  |  |  |  |  |  |  |
| *Neuroticism* | .06* | .10* | .08* | .06* | .06* | .06* | .09* | .07* | .06 |
| *Agreeableness* | .02 | .03 | .00 | -.03 | .01 | -.01 | -.01 | .02 | -.01 |
| *Conscientiousness* | -.07* | -.06* | -.03 | -.01 | -.00 | .01 | -.04 | -.04 | -.02 |
| *Extraversion* | .01 | .01 | .02 | -.03 | .00 | .01 | -.01 | .03 | .04 |
| *Open to experience* | .07* | .08* | .04 | .07* | .02 | .01 | .10* | .07* | .04 |
|  |  |  |  |  |  |  |  |  |  |
| *Demog controls* |  | X | X |  | X | X |  | X | X |
| *Identity controls* |  |  | X |  |  | X |  |  | X |
|  |  |  |  |  |  |  |  |  |  |
| *Adjusted R^2^* | .06 | .08 | .13 | .03 | .07 | .09 | .04 | .08 | .12 |
| *N* | 1515 | 1515 | 1515 | 1986 | 1986 | 1986 | 1515 | 1515 | 1515 |

Note: * p<0.05. Standardized coefficients. Demographic controls: sex; educational qualifications; race; occupational class; household income quintiles; union membership; housing tenure and religiosity. Identity controls: Remainer/ Leaver for Brexit identity and Conservative/ Labour/ Liberal Democrat for party identity.

**Appendix 11 – Experimentally manipulating the positive or negative context**

In a separate section of the questionnaire to the main survey, we split the sample into two and either induced a positive focus on the partisan in-group or a negative focus on the partisan out-group. The aim was to experimentally vary the context in which respondents found themselves. To change this context, we asked respondents who were Conservative or Labour partisans to either list positive words about their own side or negative words about the other side. After this treatment, we asked a reduced version of the positive and negative partisanship question batteries.

Respondents were randomly split into two groups. Group one were given the following question with the aim of providing a context which focuses on the positive elements of an in-group identity:

*What positive words would you use to describe [Conservative/ Labour] supporters like you? Please write down as many positive words as you can which you think accurately describe [Conservative/ Labour supporters like you.*

The party named was matched to their earlier mentioned partisan affiliation. Respondents without a partisan affiliation to the Conservatives or Labour were not included. A large box was shown to encourage longer open-ended responses.

Group two were given a different question with the aim of providing a context which focuses on the negative elements of an out-group identity:

*What negative words would you use to describe [Conservative/ Labour] supporters? Please write down as many negative words as you can which you think accurately describe [Conservative/ Labour] supporters.*

The party named here was the opposite of their earlier partisan affiliation. Respondents without a partisan affiliation to the Conservatives or Labour were not included. A large box was shown to encourage longer open-ended responses. Compliance rates for the tasks were 80 per cent for Conservative partisans and 89 per cent for Labour partisans. Levels of narcissism do not appear to have affected compliance: there is no statistically significant difference in narcissism between respondents who completed the task and those who did not.

All respondents were then given an abbreviated question battery of the positive and negative partisanship measures as below:

Positive partisanship

*When I speak about the [in-group] side, I usually say ‘we’ instead of ‘they’*

*When people criticize the [in-group] side, it feels like a personal insult*

*I have a lot in common with other supporters of the [in-group] side*

Negative partisanship

*When people criticize the [out-group party], it makes me feel good*

*I do not have much in common with [out-group party] supporters*

*I get angry when people praise [out-group party]*

The response options were ‘strongly disagree’ to ‘strongly agree’ which are scored 1–5 and then averaged for each three item battery. Question order was randomized.

Table A11 shows the results of our experiment. Here we model the effects of admiration and rivalry separately on positive partisanship and negative partisanship. We include a dummy for treatment (negative out-group focus versus positive in-group focus) and an interaction between treatment and the two aspects of narcissism.

Table A11: *How positive in-group or negative out-group focus changes the effect of admiration and rivalry on positive and negative partisanship*

|  | *Positive partisanship* | *Negative partisanship* |
| --- | --- | --- |
|  | *B* | *B* |
|  |  |  |
| Admiration | .13* | .02 |
| Rivalry | .04 | .25* |
|  |  |  |
| Negative out-group focus treatment | .08 | .04 |
| Positive in-group focus treatment | - | - |
|  |  |  |
| Admiration*Negative treatment | -.15* | -.10 |
| Rivalry* Negative treatment | .15* | .05 |
|  |  |  |

*Note:* *p<0.05. N=767. Models include controls for the Big Five personality traits and party identity. Only respondents who completed the treatment task are included.

As Table A11 shows, context affects how admiration and rivalry are associated with greater positive partisanship. In a negative out-group context, rivalry is important, but admiration has no effect on positive partisanship, but in a positive in-group context the opposite is true: admiration predicts positive partisanship, but rivalry does not. For example, for people in the positive in-group treatment, moving from a low score on the admiration aspect (1) to a high score (4) increases positive in-group attachment by 0.42 points on the 1-5 scale; whereas moving from a low score on the rivalry aspect (1) to a high score (4) only increases in-group attachment by 0.11 points. Conversely, for people in the negative out-group treatment, moving from a low score on the admiration aspect (1) to a high score (4) has an almost zero effect on in-group attachment; whereas moving from a low score on the rivalry aspect (1) to a high score (4) increases in-group attachment by 0.56 points. Although the coefficients are in the same direction, we do not find any statistically significant interactions between personality and treatment for negative partisanship. Here rivalry dominates, no matter the context.
